# Investigation of memory-enhancing effects of *Streptococcus thermophilus* EG007 in mice and elucidating molecular and metagenomic characteristics using nanopore sequencing

**DOI:** 10.1038/s41598-022-14837-z

**Published:** 2022-08-02

**Authors:** Hyaekang Kim, Soomin Jeon, Jina Kim, Donghyeok Seol, JinChul Jo, Seoae Cho, Heebal Kim

**Affiliations:** 1 Department of Agricultural Biotechnology and Research Institute of Agriculture and Life Sciences, Seoul National University, Seoul, 08826 Republic of Korea; 2 eGnome, Inc, Seoul, Republic of Korea

**Keywords:** Industrial microbiology, Computational biology and bioinformatics

## Abstract

Over the past decades, accumulating evidences have highlighted the gut microbiota as a key player in the brain functioning via microbiota–gut–brain axis, and accordingly, the beneficial role of several probiotic strains in cognitive ability also have been actively investigated. However, the majority of the research have demonstrated the effects against age-related cognitive decline or neurological disease. To this end, we aimed to investigate lactic acid bacteria strains having beneficial effects on the cognitive function of healthy young mice and elucidate underlying characteristics by carrying out nanopore sequencing-based genomics and metagenomics analysis. 8-week consumption of *Streptococcus thermophilus* EG007 demonstrated marked enhancements in behavior tests assessing short-term spatial and non-spatial learning and memory. It was revealed that EG007 possessed genes encoding various metabolites beneficial for a health condition in many aspects, including gamma-aminobutyric acid producing system, a neurotransmitter associated with mood and stress response. Also, by utilizing 16S–23S rRNA operon as a taxonomic marker, we identified more accurate species-level compositional changes in gut microbiota, which was increase of certain species, previously reported to have associations with mental health or down-regulation of inflammation or infection-related species. Moreover, correlation analysis revealed that the EG007-mediated altered microbiota had a significant correlation with the memory traits.

## Introduction

It is well established that the gut microbiota participates in various physiological processes of the host, and with an increasing number of studies, it is now becoming evident that the gut microbiota also contributes to the host behavior, emotional, and brain function^1,2^. Over the past decades, many clinical and preclinical studies have discovered the links between the gut microbiota dysbiosis and unstable mental health or central nervous disorders such as anxiety, depression^3,4^, stress susceptibility^5,6^, autism spectrum disorder (ASD)^7–10^, schizophrenia^11^, Parkinson’s disease^12–14^, and Alzheimer’s disease (AD)^15,16^, leading to the concept of microbiota-gut-brain (MGB) axis. The MGB axis refers to complex bidirectional communication between intestinal microbiota, the gut, and the central nervous system (CNS)^17^. With the current limited understanding, mechanisms underlying the interaction involve endocrinological, immunological, and neural mediators, such as that through vagus nerve signaling^17^. Several metabolites produced by bacteria such as short-chain fatty acids (SCFA) and amino acid metabolites have been demonstrated to act as neuroactive compounds, and some bacteria are discovered to be capable of direct production of neurotransmitters (e.g., dopamine, serotonin, and gamma-amino butyric acid (GABA))^18^. Such bacteria with psychotropic functions that influence and benefit the CNS of the host is now called psychobiotics^19^. Accordingly, the development of psychobiotics has been considered as an interesting strategy to improve brain health, and to date, several studies have demonstrated supplementation of specific probiotics strains, mainly *Lactobacilli* and *Bifidobacterium*, exerted positive effects on the emotional or behavioral functioning along with modifications of gut microbiota and certain neuroactive compounds level^20–23^. For instance, in some studies using disease or stress-induced rodents, bifidobacterial strains reduced anxiety-like behaviors by increasing brain-derived neurotrophic factor (BDNF) expression in the hippocampus^20,21^. Also, supplementation of *Lactobacillus kefiranofaciens* showed anti-depressant effects in CUMS mice by increasing beneficial gut bacteria and circulating TRP and splenic IL-10 levels^23^. The anxiolytic effects of *Lactobacillus rhamnosus* strains and the effects on the GABAergic system also have been actively surveyed in humans and animals of various conditions^22,24–26^.

Cognitive ability is one of the critical factors for physical and psychological well-being^27,28^. However, the majority of published studies on the cognition-enhancing effects of probiotics have focused only on pathological or old-aged populations^29–34^ since the development of agents effective for age-related cognition decline or neurological disease has become a primary scientific challenge^35,36^. Though, enhancement in cognitive performance can be important as well for children and young adults who are relatively more exposed to the cognitively demanding environment^37,38^. As it was demonstrated that higher cognitive functions in those young ages are positively correlated with higher academic achievements^38–40^, enhanced cognitive skills such as memory and learning ability is generally recognized as a highly desirable function for those ages. With respect to the cognitive ability, it was shown that *Lactobacillus paracasei* K71 prevented age-related cognitive declines in senescence-accelerated mice^29^, and administration of *Lactobacillus casei* LC122 and *Bifidobacterium longum* Bl986 improved learning and memory ability in aged mice, accompanied by alteration of gut microbiota^30^. Also, elderly people supplemented with *Bifidobacterium bifidum* BGN4 and *B.* longum BORI showed improved cognitive function and mood by significantly reducing proinflammatory microbiota (*Eubacterium*, *Allisonella*, *prevotellaceae*) and increasing serum BDNF level^33^. Moreover, combined administration of *B. bifidum* TMC3115 and *Lactobacillus plantarum* 45 improved spatial memory impairment and gut dysbiosis in the transgenic mouse model of AD^34^. However, it is currently unclear whether they could exert similar positive effects in healthy young individuals as well. To this end, we aimed to investigate lactic acid bacteria strains having positive effects on the cognitive function of healthy young mice and elucidate underlying probiotics and psychobiotics properties by carrying out genome and gut microbiota community analysis.

Unraveling the correct composition and functionality of gut microbiota is an essential step for studying interactions of the MGB axis. However, most of the previous studies have been performed through targeting only a few hypervariable regions of the 16S rRNA gene (e.g., V3–V4, V4–V5, V4–V6) to infer taxonomy since the most commonly used short-read based sequencing platforms allowed length of only partial regions (~ 100–500 bp)^41–43^. This approach would be feasible to capture the global structure but has critical limitations as it only gives family- and, in some cases, genus-level taxonomic resolution^41,44^. Furthermore, the different region selection could lead to different classification results or different taxonomic resolution^41,45^. Fortunately, these strategies have been gradually replaced by the long-read sequencing technology such as PacBio or Oxford Nanopore Technologies which provides reads that can span the full 16S rRNA gene (~ 1500 bp) or even the nearly-entire rRNA operon (~ 4300 bp; 16S rRNA gene-ITS region-23S rRNA gene)^44–46^. These markers have been successfully characterizing various bacterial communities down to species level, including mouse gut microbiota^45–48^. It was demonstrated that both longer markers provided higher and more accurate taxonomic resolution than the short-read sequencing data, particularly for the key members of the gut microbiota such as *Bifidobacterium animalis* and *Bifidobacterium pseudolongum*^44,46^, but 16S–23S operon provided remarkably improved taxonomic assignment at the species level due to the increased number of informative sites^45^. Based on the advantages, here we utilized 16S–23S rRNA operon amplicon sequencing to explore potential psychobiotics-mediated impacts on the gut microbial composition at the species level, using Nanopore Flongle which is ideal for microbial sequencing. Some earlier associative studies have been performed to define gut microbiota features associated with the psychological and cognitive status of the hosts^30,34,49–52^, but given the limited taxonomic resolution, the present study would provide more accurate species-level profiling and even identify discover previously unreported microbes potentially related to the cognitive ability.

**Figure 1:**
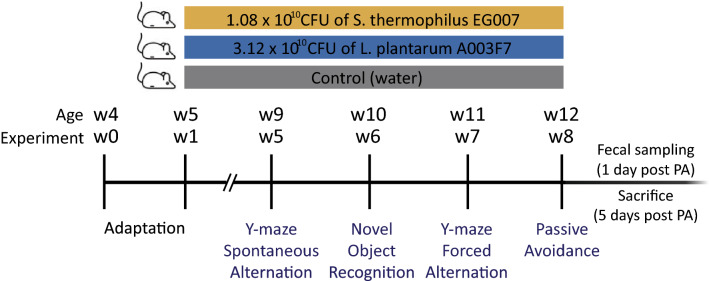
Schematic diagram of study design. Male SPF C57BL/C mice (n = 36) were randomly divided into three groups, and each group was supplemented with *S. thermophilus* EG007, *L. plantarum* A003F7, and water (control). From week 5 (nine weeks of age) onward, all mice were subjected to the same series of behavior tests to assess their cognitive performance, the Y-maze test (spontaneous alternation and forced alternation) for spatial memory, the novel object recognition test for nonspatial memory, and the passive avoidance test for long-term associative memory. After 1 day following the last test, fecal samples were collected for gut microbiome community analysis, and all mice were sacrificed after 5 days.

In the present study, we assessed the effects of two probiotic strains administration, *Streptococcus thermophilus* EG007 (EG007) and *L. plantarum* (A003F7), on various aspects of cognitive abilities (short term spatial and non-spatial, and long-term associative memory) (Graphical experiment design is presented in Fig. 1). Our results suggested that the EG007 had spatial and non-spatial memory and learning-enhancing effects on healthy 5–12 weeks old male mice. Investigation of the probiotics and psychobiotics characteristics by constructing complete genome sequence of EG007 identified genes encoding various metabolites beneficial for a health condition in many aspects, including GABA producing system. Further, by 16S–23S sequencing of gut microbiota, EG007-derived positive impacts on the composition and functional profile of gut microbiota were detected at the species level and a more detailed microbiota-cognition relationship was identified, which may influence gut-brain communication and improve cognitive abilities. As far as we know, this is the first study examining the effects of a single *S. thermophilus* strain on brain function and identifying associated microbial alteration at the species level. These results will provide insights into promising psychobiotics characteristics of *S. thermophilus* EG007 with possible MGB axis modulating capability, which can be applicable for a population of healthy and younger people.

## Results and discussion

### Effects of *S. thermophilus* EG007 and *L. plantarum* A003F7 on learning and memory of young healthy mice

#### General effects of probiotics

Each mouse has consumed an average of 1.08 × 10^10^ CFU of *S. thermophilus* EG007 (ST group) or 3.12 × 10^10^ CFU of *L. plantarum* A003F7 (LP group) per day throughout the whole study (8 weeks) (Supplementary Fig. 1). The average body weight during the course of the experiment is shown in Supplementary Fig. 1. The curve of body weight gain for the LP group is constantly under two other groups on the graph, but there was no significant difference between treatments for the total body weight gain (week0–week8) (one-way ANOVA, *P* = 0.494). Also, we did not observe any abnormal behavior nor subject mortality during the probiotic supplementation period. Thus, we could conclude that the consumption of both probiotics had no adverse effect on overall health.

#### Y-maze spontaneous alternation test

**Figure 2:**
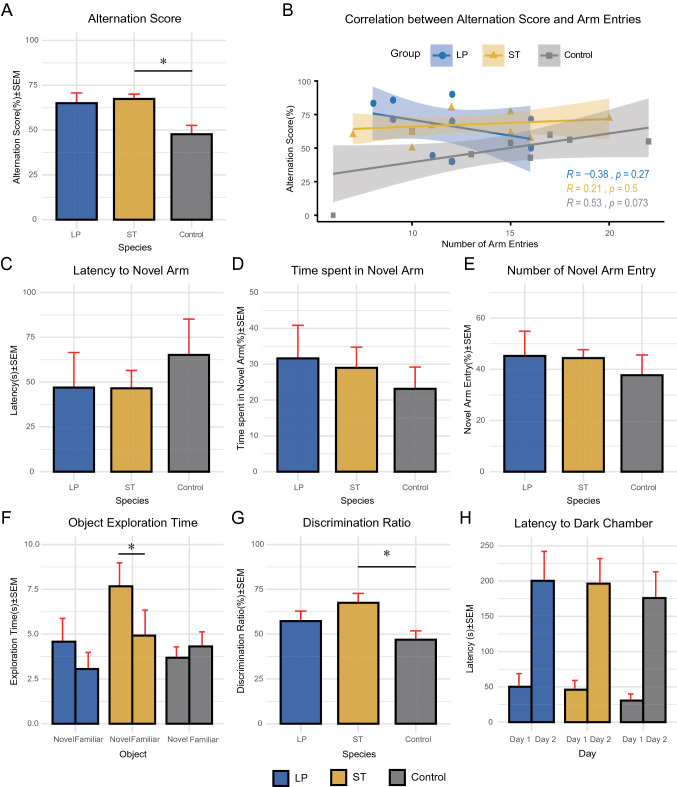
Cognition-related behavior tests. (**A,B**) Alternation score (%) and the correlation between the alternation score and a total number of arm entries of Y-maze spontaneous alternation test. (**C–E**) Latency to novel arm (s), time spent in the novel arm (%), and a total number of novel arm entries (%) in Y-maze forced alternation test. (**F,G**) Time spent in exploration of novel and familiar objects (s) and discrimination ratio (%) in the novel object recognition test. (**H**) Latency to dark chamber (s) on day1 (training session) and day 2 (testing session) of passive avoidance test. All data were expressed as mean ± standard error of the mean (SEM). Differences between the mean values were tested using one-way analysis of variance (ANOVA) or Kruskall-wallis test, where appropriate, followed by the Tukey’s *post-hoc* test. Correlations were assessed by Pearson’s statistics. The difference was considered statistically significant when *P*-value < 0.05 (indicated with *).

The effects of EG007 and A003F7 on the short-term spatial working memory and exploratory behavior enhancement were evaluated using Y-maze spontaneous alternation after 5 weeks of administration. In this test, mice were allowed to freely explore three arms, and the number of total arm entries and alternation score were calculated. In the spontaneous alternation score, both LP and ST groups showed the enhanced ability to discriminate between maze arms compared with the control group. As shown in Fig. 2, a significant effect of treatment on the alternation score was observed (67.4 ± 2.72% vs. 65 ± 5.7% vs. 47.7 ± 4.93% for ST, LP, and Control, Kruskal–Wallis, *P* = 0.00956), with *post-hoc* analysis revealing that the ST group exhibited significantly improved short-term spatial memory (Tukey, *P* = 0.0354). We did not observe any significant differences between LP mice and control mice. The number of total arm entries was similar across all experimental groups, showing no difference in the general locomotor activity. And there was no correlation between the number of total arm entries and the alternation score (Pearson’s correlation, r = 0.16, *P* = 0.18), indicating that any differences in locomotor activity did not influence the alternation score (Fig. 2). These results suggest that the administration of *S. thermophilus* can improve spatial learning and memory in healthy young mice.

#### Y-maze forced alternation test

After 7 weeks of administration, the forced alternation test was performed. For latency before entering the first novel arm (Fig. 2), the control group showed delayed entry into the novel arm, compared to the probiotics-treated groups, although the differences were not statistically significant (47 ± 19.6 s vs. 46.6 ± 9.94 s vs. 65.2 ± 20 s for ST, LP, and Control, Kruskal–Wallis, *P* = 0.7761). Regarding the percent time spent in the novel arm over the other arms, both ST mice and LP mice showed a higher percentage than the control group (29 ± 5.76% vs. 31.6 ± 9.22% vs. 23.1 ± 6.06% for ST, LP, and Control), but there were no statistically significant differences (one-way ANOVA, *P* = 0.666). Consistent with this, the percentage of entries into the novel arm over the other arms for both probiotics-fed groups were higher compared to that for the control group, with no significant differences between the groups (44.4 ± 3.29% vs. 45.2 ± 9.64% vs. 37.7 ± 7.89% for ST, LP, and control, Kruskal–Wallis, *P* = 0.9913).

#### Novel object recognition test

The novel object recognition test was conducted to test short-term nonspatial working memory in mice after 6 weeks of probiotics administration. In this test, the memory performance was assessed by measuring the discrimination ratio. When comparing the total time of investigation of both objects, Fig. 2 shows that mice in all experimental groups tended to interact more with the novel object than the familiar one during the testing phase. Both groups fed with either EG007 and A003F7 showed an increase in discrimination ratio compared to the control group (67.5 ± 5.26% vs. 57.3 ± 5.62% vs. 46.9 ± 4.89% for ST, LP, and Control; Fig. 2). There was a significant difference between groups (one-way ANOVA, *P* = 0.03), and *post-hoc* analysis indicated that only ST mice had significantly increased preference for the novel (Tukey, *P* = 0.0264). These results indicated that the administration of *S. thermophilus* can help enhancing the ability to discriminate between familiar and novel objects through improving nonspatial memory skills in healthy C57BL mice.

#### Passive avoidance test

After 8 weeks of administration, the fear-motivated passive avoidance test was performed to evaluate the long-term associative memory of mice. In this test, memory performance was indicated by the latency to enter the dark chamber where the moue had experienced a foot shock. Therefore, more increased latency indicates better memory retention. As shown in Fig. 2, in day 1, mice readily entered the dark chamber and showed similar behavior toward it, and there were no differences in the latencies between groups (46 ± 13.1 s vs. 50.2 ± 18.4 s vs. 30.5 ± 9.41 s for ST, LP, and Control, Kruskal–Wallis, *P* = 0.3684). During the testing phase, 24 h later, latencies for all of the groups increased to 196 ± 35.5 s, 200 ± 41.7 s, 176 ± 37.2 s (ST, LP, and Control, respectively), indicating the formation of the contextual memory, but no significant differences were shown between the groups (Kruskal–Wallis, *P* = 0.969).

In summary, a series of behavior tests assessing different aspects of cognitive skills indicated EG007 significantly increased short-term spatial (SA) and non-spatial (NOR) learning and memory in healthy young C57BL/C mice. So far, the influence of *S. thermophilus* on the host CNS function has only been investigated in forms of probiotics mixture or yogurt in few studies^53,54^, and this is the first study to examine the effects of a single *S. thermophilus* strain. Although previous studies observed some *L. plantarum* strains were capable of relieving opposition/defiance behaviors in children with ASD^55^ or attenuating cognitive impairments in the AD mouse model^56^, A003F7 did not show significant enhancements in this study.

### Constructing complete genome of *S. thermophilus* EG007 and characterization of general genome features

In order to determine the genomic characteristics of EG007, we constructed the complete genome of *S. thermophilus* EG007. Flongle sequencing using 20 K insert size library generated 106,853 raw reads, totaling approximately 1.0Gbp of sequences (529.14x coverage) (Table 1). After removing adapter sequences and chimeric reads using Porechop, reads with a quality score below 10 were discarded using Nanofilt, and 77,148 reads (a total of 737.7 Mb) remained. Reads had an average length of 9562.56 bp and N50 length of 14,953 bp, and they were subjected to genome assembly using CANU. As shown in Table 1, CANU successfully generated 2 contigs, with a total length of 1,901,104 bp, including contigs of 1,894,187 and 6917 bp. In order to reduce insertion/deletion errors which could affect the gene prediction, multiple rounds of polishing with racon and medaka were conducted until no additional correction could be made. Polishing steps reduced the number of pseudogenes from 435 to 218 and resulted two contigs of 1,895,271 bp and 6932 bp, and the smaller contig was confirmed as a plasmid using Plasflow. Lastly, the assembled reads were circularized using Circlator, and the completed genome of EG007 consisted of a circular chromosome 1,860,782 bp in size and a 3500 bp plasmid with 39.05% of GC contents, which were in range with other available *S. thermophilus* strains.

**Table 1:** Summary statistics for the assembly of *S. thermophilus* EG007 complete genome.

	Raw reads	Trimmed reads	Filtered reads (q > 10)
Reads	106,853	106,813	77,148
Bases (Mb)	1005.36	1000.78	737.71
Coverage	529.14x	526.72x	388.27x
Average read length	9408.79	9369.42	9562.56
N50	15,032	15,021	14,953

Prokka annotation indicated that the genome contained 2118 genes (CDS: 1952, misc_RNA: 80, rRNA: 18, tRNA: 67, tmRNA: 1) including two genes (replication protein and type 1 restriction enzyme protein) located in the plasmid. The general genomic structure of the EG007 is depicted in Supplementary Fig. 2. The predicted CDSs were then assigned in clusters of orthologous group (COG) functional categories (Supplementary Fig. 3). 1554 proteins were classified into 19 specific COG categories, and the majority of them were involved primarily in housekeeping and metabolic processes. Among them, translation, ribosomal structure and biogenesis (J) and DNA replication, recombination and repair (L) represented the two most abundant categories. Also, an inspection of gene contents in metabolism revealed that EG007 seemed biased towards four categories: amino acid (E), inorganic ion (P), carbohydrate (G), and nucleotide (F) transport and metabolism, which represents 32% of assigned proteins.

Analysis using resfinder, CARD-rgi, and VFDB indicated that EG007 neither contain any antibacterial resistance genes nor virulence-related genes, whereas some strains of *S. thermophilus* were reported to possess resistance genes to tetracycline or erythromycin^57^. Also, 13 genomic islands (named from GI 1 to GI 13) (Supplementary Fig. 2, Supplementary Table 1) were predicted in the chromosome by Islandviewer 4, suggesting evidence of lateral DNA acquisitions^58^. In addition, CRISPRCasFinder detected three distinct CRISPR/Cas loci (CRISPR1, CRISPR2, and CRISPR3), similar to other *S. thermophilus* strains^59^. CRISPR-Cas system is widely distributed among prokaryotes as an adaptive immune system against bacteriophage infection. However, CRISPR1 of EG007 contained the highest number of spacers (61) among the *S. thermophilus* strains analyzed so far, given that the number of spacers of known *S. thermophilus* CRISPR1 varied between 11 and 42^60^. This suggests a possible effective defense mechanism of the CRISPR1 against different bacteriophages compared to those of other sequenced *S. thermophilus* strains, thereby conferring a better adaptive immunity. Consistent with these, five incomplete prophage-like regions were identified in the genome which probably are traces of the ancient phage infections, followed by inactivation and slow decay^61^, indicating no recent infection occurred.

### Identification of genes associated with psychobiotics potential in *S. thermophilus* EG007

We further investigated the functionality of the strain as probiotics and potential psychobiotics. First, for bacterial strains to be effective as probiotics, the ability to survive through the gastrointestinal (GI) tract environment is a key factor. Genomic analysis of EG007 showed that it presented coding sequences that possibly confer resistance abilities to various stress. A total of 24 genes that lead to acid tolerance (9 genes), Oxidative stress tolerance (11 genes), and bile salt tolerance (1 gene) were detected in the genome (Supplementary Table 1). Three genes associated with strain’s adhering ability were also found, which was an important feature for successful colonization in the GI tract after intake. In addition, the genome showed the presence of extracellular polymeric substance (EPS) genes cluster which was involved in the extracellular polysaccharide synthesis (Supplementary Table 1). The presence of EPS genes gives probiotics important industrial properties related to the desired texture or reduced syneresis of fermented dairy products and health-promoting properties such as antimicrobial activity, anti-inflammatory activity, and immunomodulation^62,63^. Moreover, it was also studied that the EPS synthesis correlated with robustness in cell aggregation, and thereby providing higher survival properties in the GI tracts^64^. The structural and compositional diversity of EPS molecules are varied by both number and the type of genes organized in the clusters, and many different kinds of clusters have been discovered in *S. thermophilus*. The current analysis revealed a relatively large size (28,057 bp) of the cluster in EG007 which were composed of 33 genes (from deoD to permease gene) including UDP-galactopyranose mutase gene, rarely found in *S. thermophilus* EPS clusters^65,66^, and interestingly, it contained several hypothetical genes and transposase genes distributed downstream in the cluster. These observations were also reported in strain JIM8232, CNRZ368, and ND03. The transposase genes were located in the GI 6. Lastly, screening of the genome using BAGEL4 revealed a total of five putative bacteriocin biosynthesis gene clusters (cluster1: streptide; cluster2: sactipeptides; cluster3: Blp family; cluster4: lanthipeptides class 1; cluster5: sactipeptides), together with genes coding for the self-immunity protein and the transport system (Supplementary Fig. 4, Supplementary Table 1). Bacteriocin-producing ability of probiotics is a key feature as these antimicrobial peptides can facilitate competition of the probiotics by directly eliminating pathogens and also serve as signaling peptides^67^. Similar to the genes in the EPS cluster, all five clusters were carried by genomic islands (cluster 1 in GI 10; cluster 2 in GI 5; cluster 3 in GI 11; cluster 4 in GI 2; cluster 5 in GI 13), and the lantibiotic biosynthesis genes (in cluster 4) showed high similarity with *Streptpcoccus dysgalactiae* (100% coverage, 97.18% identity) and *Streptococcus pneumoniae* (98% coverage, 80.28% identity). These results indicate that there might have been horizontal gene transfers (HGT) events of EPS and bacteriocin clusters, and it provided EG007 beneficial traits for improved adaptability in the GI environment and probiotic functionalities.

**Figure 3:**
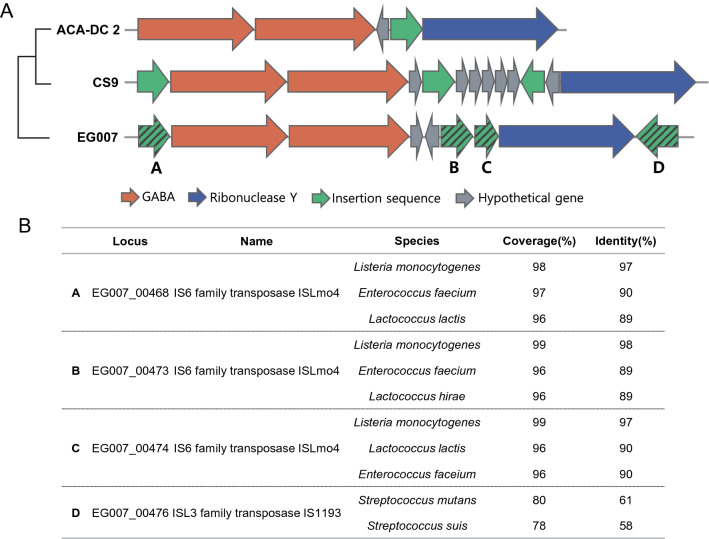
Gene organization around GABA producing operon. (**A**) Varied organization around GABA gene operon among *S. thermophilus* EG007, CS9, and ACA-DC-2. Genes encoding the related proteins are marked with a different color. (**B**) Similarity search results of insertion sequences flanking GABA gene operon of EG007.

It is well known that a large proportion of neurotransmitters such as serotonin, dopamine, and GABA is produced in the gut^68^. Several studies confirmed that lactic acid bacteria were one of the most important neurotransmitter producers among the enteric microbes, and some of *S. thermophilus* strains with GABA-producing ability have also been discovered. GABA is a bioactive molecule that exerts several beneficial physiological functions in humans and animals^69^. The biosynthesis of GABA by enteric microbes is performed by the glutamate decarboxylase (GAD) system, which is composed of the GAD enzyme (encoded by *gadA* or *gadB*) and glutamate/GABA antiporter *GadC*^70^. Once *GadC* transports l-glutamate into a cell, it is decarboxylated by GAD enzyme to form GABA^70^, and the decarboxylated product GABA is exported back to the extracellular matrix through *GadC*^70^. Generally, the microorganisms with the GAD gene are known to be capable of synthesizing GABA^71^. Genome analysis of EG007 revealed that the strain possessed entire *gadB*-*gadC* genes (1380 and 1434 bp, respectively) in its genome (Fig. 3). The translational product (459 amino acid protein) of the *gadB* gene was identical to that of strain APC151 with known GABA synthesis ability. The survey of 65 publicly available complete genome sequences of *S. thermophilus* found the presence of the genes in only 18 strains, and the genetic organizations around the GAD systems presented a high variability for the same species (Fig. 3, Supplementary Fig. 5). Interestingly, in EG007, the *gadB*-*gadC* genes were flanked by multiple transposases elements (three IS6 family transposase ISLmo4 and an ISL3 family transposase IS1193), and the genetic organization was closest to the strain EU01 among the 18 strains. Similarity searches of the transposase elements against ISFinder DB showed near identity with the known insertion sequences of *Listeria monocytogenes*, *Enterococcus faecium*, and *Lactococcus lactis* (Fig. 3). It implies that an ancestral strain of EG007 may have originally acquired the operon by HGT from other GABA-producing microorganisms. According to previous findings, it has been reported that strains from *L. monocytogenes*, *L. lactis*, *E. faecium* were capable of producing GABA^72–74^. Furthermore, since microorganisms are able to raise the cytoplasm and extracellular pH by exporting the produced GABA, it is speculated that having the GAD system can be also beneficial for the microbe itself, by allowing them to survive better under acidic conditions^75^.

GABA plays an important part in mood and stress response by acting as a major inhibitory neurotransmitter in the mammalian central nervous system^76–78^. In addition to that, recent researches have assessed its essential role in cognitive functioning in animals and humans. For instance, Auger et al. revealed that the prefrontal GABA hypofunction severely disturbed spatial reference and short-term memory in rats^79^, and in humans, it was found that the GABAergic levels in the dorsal anterior cingulate cortex were significantly decreased in patients with schizophrenia and psychotic disorder^80^. In studies using rats, oral intake of GABA increased hippocampal GABA level and enhanced learning and memory performances, which was confirmed by measuring NOR, maze, and PA test^81,82^. These are in agreement with the present results that the indices of spatial (Y-maze SA) and non-spatial working memory (NOR) processes were improved following supplementation of EG007. Moreover, in recent studies by Yunes et al. and Patterson et al., it was demonstrated that ingestion of GABA-producing LABs had positive effects on depressive-like behavior in the mice model^76,78^. As shown in previous studies, GABA receptors present on the enteric neurons of the GI tract, and the expression of the receptors are increased upon GABA-producing probiotics consumption. It is assumed that the GABA produced in the GI tract is able to directly modulate the host GABAergic signaling process using the enteric GABA receptors and vagus nerve as a route of communication^17,83–85^. We, thus, presumed that EG007 could have direct effects on the cognitive ability enhancement of the mice through influencing the CNS via the gut-brain axis.

In summary, EG007 possessed genes encoding various bioactive molecules beneficial for modulating host health in many aspects, and by screening antibiotic resistance and virulence-related genes, we also concluded that it can be considered safe as none of those genes were detected in the genome. We found evidences in the genome of EG007 that the ancestral strain might have undergone HGT to obtain genes related to the EPS, bacteriocin, and GABA productions which could also increase fitness in the GI tract. These acquisitions, consequently, might contribute to enhance probiotic functionalities. Especially, as one of the rare GAD system-possessing strains among *S. thermophilus*^86^, EG007 can be a great candidate to serve as next-generation probiotics with possible gut-brain axis modulating capability. Similar to the preceding studies, GABA produced by EG007 might have directly promoted the cognitive ability of ST supplemented mice group by stimulating enteric GABA receptors.

### Nanopore sequencing of 16S–23S operon and identification of microbial alterations of EG007 treated mice

To investigate whether EG007-related alteration in the intestinal microbiome was a component of memory enhancement, we compared and analyzed the gut microbial communities of EG007 and control groups. In general, for taxonomic classification of the microbial community, only a few hypervariable regions of the 16S rRNA gene have been utilized as a taxonomic marker, but it is known that these limited regions classify only to family- or genus-level and gives varied results depends on the region selections^41–45^. To take advantage of long-read sequencing technologies, an increasing number of studies are attempting to apply longer regions of markers such as full-length 16S and 16S–23S rRNA which allow higher and more accurate resolution of the microbiota^45–48^. In this study, the 16S–23S rRNA region (approximately 4200 bp) of the ribosomal operon was chosen as the marker to obtain increased resolution at the species level^45^. We randomly collected eight fecal samples from control and ST group mice on the 57th day of the experiment. Amplification of 16S–23S rRNA genes from the isolated metagenomic DNAs was achieved using specific primers, and nanopore sequencing libraries were constructed from these amplicons. In order to avoid read loss during demultiplexing step, we performed eight separate sequencing runs for each of the samples instead of multiplexing (1 fecal sample library per 1 Flongle flow cell). Flongle sequencing runs yielded a total of 2,624,400 pass reads, with an average of 321,513 reads/sample for the control group and an average of 334,588 reads/sample for the ST group (Table 2). Following adapter trimming and filtering by read length, a total of 1,058,700 and 1,100,958 reads were retained (82.32% and 82.26% of pass reads), with an average read length of 4119.84 bp and 4121.25 bp for the control and the ST group, respectively, which were nearly the full length of the 16S–23S rRNA gene (about 4200 bp) (Table 2).

**Table 2:** Summary statistics for 16S–23S operon sequencing of the gut microbiome of the ST and control group.

		Total reads			Pass reads		Trimmed reads			Filtered reads (3500–5000 bp)	
		Reads (K)	Estimated base (Mb)	N50 (K)	Reads (K)	N50	Reads (K)	N50	Average read length (bp)	Reads (K)	Average read length (bp)
ST group	S1	188.7	874	4.88	186.7	4137	186.7	4137	3897.49	160.4	4133.78
	S2	521.6	241.0	4.98	513.7	4122	513.7	4122	3841.20	435.6	4122.67
	S3	270.7	124.0	4.89	266.3	4136	266.2	4136	3867.40	225.7	4138.00
	S4	357.4	158.0	4.74	352.7	4104	352.7	4104	3785.95	279.2	4090.54
Control group	W1	400.5	179.0	4.9	395.8	4139	395.8	4139	3730.65	312.9	4128.71
	W2	291.4	129.0	4.69	288.0	4113	288.0	4113	3847.10	248.5	4111.76
	W3	175.0	728	4.76	172.4	4107	172.4	4107	3827.51	147.2	4096.70
	W4	419.2	188.0	4.68	411.0	4133	410.9	4133	3881.67	350.1	4142.15

Next, we assigned taxonomy of these processed reads by aligning to MIrROR DB using minimap2. The MIrROR DB which was developed in our accompanying study contains 97,781 16S–23S rRNA sequences of 43,653 bacterial strains representing 9485 different species. With this method, 92.85% (982,957 of 1,058,700) and 94.48% (1,040,266 of 1,100,958) reads were aligned to the taxonomic reference sequences, with average values of alignment block length of 4299.5 and 4292.5 bp and AS score of 3044.75 and 3122 for the control and ST group, respectively (Table 3). We were able to detect a total of 1090 taxonomic units at the species level, with an average of 607 species and 631 species were detected for the control and ST group, respectively. In beta diversity analysis measured by PCoA for both weighted and unweighted UniFrac metrics and nMDS based on Bray–Curtis dissimilarity, subtle separation by groups was observed, even though Permutational Multivariate Analysis of Variance (PERMANOVA) failed to detect a significant difference (Supplementary Fig. 6). Likewise, alpha-diversity was not significantly affected by EG007 supplementation as estimated by several indices (Chao-1, ACE, Observed, Simpson’s, and Shannon’s). The composition of gut microbial communities at hierarchical taxonomy levels from the two groups were presented in Supplementary Fig. 7. The predominant phyla were Bacteroidetes, Firmicutes, and Actinobacteria, accounting for more than 95% together. At the genus level, *Muribaculum* was the most abundant genus, followed by *Lactobacillus*, *Bacteroides*, and *Alistipes*, similar to the previous mouse gut microbiota analysis^87–89^, and 163 common genera were characterized.

**Table 3:** Summary of taxonomic assignment results of reads generated using 16S–23S operon sequencing.

		Taxonomic assignments using MIrROR DB									
		Read counts	Reads assigned to species level (coverage %)	Species	Number of matching bases in the mapping			Number of bases, including gaps, in the mapping			AS score
					Min	Avg	Max	Min	Avg	Max	Avg
ST group	S1	160,429	151,607 (94.50%)	651	1529	3362	4515	2500	4301	6356	2942
	S2	435,596	416,274 (95.56%)	623	1542	3416	4548	2500	4301	6221	3287
	S3	225,694	219,829 (97.40%)	606	1531	3440	4583	2500	4290	6226	3468
	S4	279,239	252,516 (90.43%)	645	1520	3319	4472	2500	4278	5417	2791
Control group	W1	312,859	282,526 (90.30%)	641	1550	3333	4455	2500	4295	5771	2789
	W2	248,542	238,989 (96.16%)	680	1509	3359	4540	2500	4290	6277	2989
	W3	147,213	137,934 (93.70%)	602	1516	3374	4438	2500	4286	5385	3067
	W4	350,086	323,508 (92.41%)	620	1558	3440	4592	2500	4327	6239	3334

**Figure 4:**
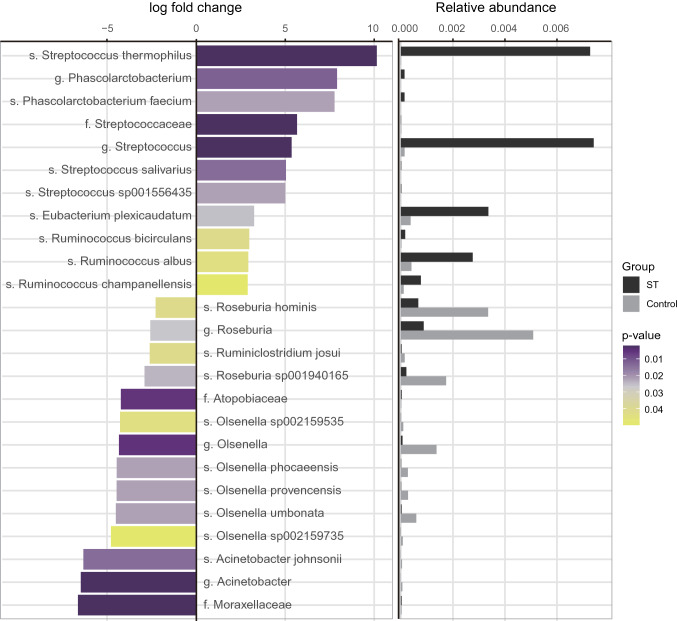
Significantly modulated gut microbiota by S. thermophilus EG007 consumption. For detection of significantly altered taxonomic units, negative binomial based generalized linear model (GLM) was employed using EdgeR with trimmed mean of M-value (TMM) normalization method, and significance was defined by *P*-value < 0.05. For each of the altered taxonomic units, log(Fold change) was shown in the left panel, and the relative abundance was presented on the right panel.

We further performed differentially abundant microbiota analysis using TMM-normalized GLM method in EdgeR package to identify microbial alterations that significantly contributed to differences between groups. We identified 25 taxonomic units across all taxonomic levels that were significantly over- or under-represented by consumption of EG007 (*P*-value < 0.05) (Fig. 4). After consumption of EG007, 11 significantly increased in abundance, and significantly lower levels of 14 taxonomic units were observed (Fig. 4). The altered species were mostly within phylum Firmicutes (genus *Roseburia*, *Ruminiclostridium, Ruminococcus, Eubacterium, Faecalibaculum*, and *Streptococcus*, and *Phascolarctobacterium*) and *Actinobacteria* (genus *Olsenella*). Abundance of *S. thermophilus*, *S. salivarius*, and *Streptococcus* sp001556435 was significantly abundant in all samples of the ST group. Overall, enriched taxonomic units were mostly composed of cellulolytic microorganisms including four *Ruminococcus* species^90,91^, three *Streptococcus* species, and *Eubacterium plexicaudatum*^90,92^, whereas the abundance of potentially pathogenic bacteria, *Acinetobacter johnsonii* and five *Olsenella* species were reduced by EG007 supplementation. *Acinetobacter* is prevalent pathogen in intensive care units, and species such as *Acinetobacter baumannii* is known to cause nosocomial infections in humans^93^. Although *A. johnsonii* is generally considered nonpathogenic, recent genome analysis of strain MB44 found potential virulent factors homologous to virulent proteins of *A. baumanii*^94^. Similarly, in previous studies, abundance of genus *Olsenella* was positively correlated to gut inflammation and STH infection, which also significantly reduced following clearance of the infections^95,96^. The reduction of these microbes might be associated with the antagonistic effect of *S. thermophilus* against various pathogens^97^ as the presence of several kinds of putative bacteriocin clusters in the EG007 genome was also identified. A number of studies have proposed the functions of probiotics in a decrease of proinflammatory microbes and increase of microbiota with anti-inflammatory activities, which might in turn prevented the pathogenesis of neurodegeneration via the MGB axis^33,98^. Interestingly, genus *Phascolarctobacterium* and *Phascolarctobacterium faecium* that increased about eightfold in ST group mice were previously reported to be positively associated with the positive mood in the human host^99,100^, and beneficial effects of *P. faecium* also have been identified in several studies^101,102^. In addition, EG007 supplementation led to significant increases in the proportion of *Ruminococcus* species. Notably, prior studies have documented the association of genus *Ruminococcus* to AD, cognitive impairment, and depression^100,103,104^. Taken together, the results imply that supplementation of *S. thermophilus* led to positive changes of gut microbiota which may contributed to the beneficial effects in improvement of cognitive ability in mice.

A recent study evaluating changes in the composition of the human gut microbiome upon yogurt consumption also observed significant increases in *S. thermophilus* and *Ruminococcus* species^105^. While a mouse microbiome catalog revealed that the human and mouse microbiota share a considerable number of microbial genera and showed significant overlap of annotated functions, it should be noted that there still exist large differences in the quantitative and qualitative representation of taxa between the two species^89,106^. Moreover, there are differences in microbial composition between mouse strains, and even mouse and human strains of the same microbial species can be very different^107^. Therefore, there are limitations to predicting responses in humans from such results in animal models. Further clinical studies involving human subjects are needed to evaluate whether the EG007 administration can induce similar alteration in the human gut microbiome and their cognitive functions.

### Associations between gut microbiota and cognition traits

In order to identify bacterial communities related to cognitive abilities, we further conducted correlation analysis between the normalized abundance of each taxonomic unit and the two improved cognition traits (Y-maze SA and NOR) via Pearson correlation analysis. 44 taxonomic units including 26 at the species level were significantly associated with spatial (Y-maze SA) and non-spatial (NOR) recognition memory (*P*-value < 0.05) (Supplementary Table 2). The differential ratio in the NOR test was correlated with 22 taxonomic units, including 15 positively correlated and 7 negatively correlated. The alteration score in the Y-maze test was correlated with 22 taxonomic units, including 13 positively correlated and 9 negatively correlated. Among them, *Pediococcus inopinatus* (r = 0.90) and *S. salivarius* (r = 0.89) had the most highly positive correlation with the differential ratio and the alteration score, respectively. It is worth noting that the well-known lactic acid bacteria, *Lactobacillus fermentum* (r = 0.73) and *P. inopinatus* displayed a significantly positive correlation with the non-spatial recognition memory, and a recent study indicated that a neuroinflammation inhibitory effects of *P. inopinatus* on glial inflammation and ameliorating ataxia symptoms of Parkinson’s disease mouse model^108^. *P. inopinatus* was also positively correlated with the abundance of *S. thermophilus* (r = 0.79). Interestingly, the abundance of *Staphylococcus aureus*, generally known as an opportunistic pathogen, was significantly negatively associated with the alteration score (r = − 0.78) while other *Staphylococcus* species (*S. schleiferi* and *S. pseudintermedius*) displayed the opposite relationship (r = 0.72, r = 0.73, respectively).

**Figure 5:**
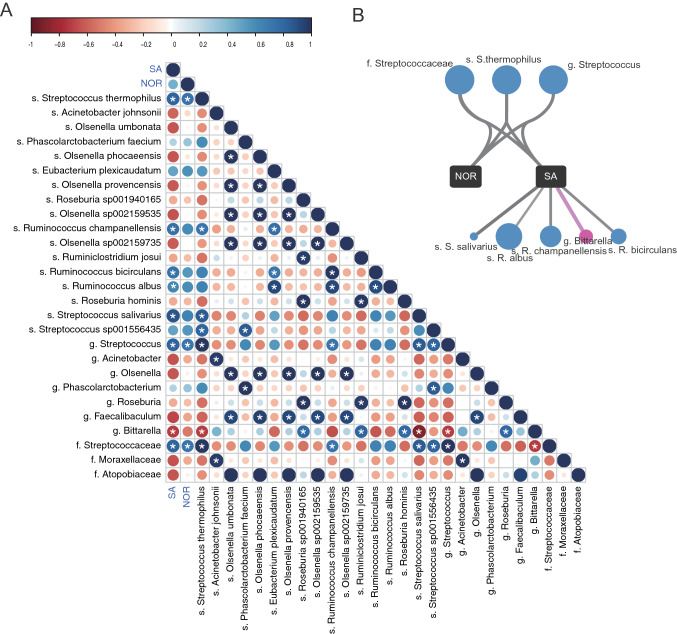
Relationship between altered gut microbiota and cognition traits. (**A**) Correlation between gut microbiota significantly modulated by EG007 consumption and cognition traits (SA and NOR). Strong correlations are represented as large circles, and weak correlations are represented as small circles. The colors of the scale bar indicate the correlation coefficient with dark blue indicating perfect positive correlation (correlation coefficient = 1) and dark red indicating the perfect negative correlation (correlation coefficient =  − 1). Statistical significance was defined as *P*-value < 0.05 (indicated with *). (**B**) Networks between the cognition traits and the significantly modulated gut microbiota which are highly correlated with the traits. Circle nodes represent taxonomic units, and rectangle nodes represent cognition traits. The sizes of circle nodes indicate the relative abundance in the EG007-treated mice gut, and the color of circle nodes represent increased (blue) and decreased (pink) after EG007 consumption. The lines represent the degree of correlation (width) and positive (grey) or negative (pink) correlation.

Among the 44 microbiota detected to have correlation with the cognition traits, we observed that 8 were also significantly altered in the ST group, indicating these taxonomic units were not only modulated by EG007 supplementation but also closely related to the cognitive ability (Fig. 5). As shown in Fig. 5, three (*S. thermophilus*, genus *Streptococcus*, and family *Streptococcaceae*) were associated with the non-spatial recognition memory, and eight (*Ruminococcus albus*, *Ruminococcus bicirculans*, *Ruminococcus champanellensis*, genus *Bittarella*, *S. salivarius*, *S. streptococcus*, genus *Streptococcus*, and family *Streptococcaceae*) were associated with spatial recognition memory. Both of the scores exhibited a positive association with the abundance of *S. thermophilus* and its higher taxonomic levels. The results showed that three *Ruminococcus* species, whose abundances were significantly enriched by EG007, were positively correlated with spatial recognition memory. *Ruminococcus* species are generally described as consistently present SCFA-producing bacteria in the healthy human gut^90,109^, and according to previous researches, the relationship between the genus *Ruminococcus* and the brain functions has been described vary widely. Some studies suggested elevated fecal *Ruminococcus* levels in children or mouse model with ASD^110–112^, while others observed decreased fecal *Ruminococcus* in ASD children compared to the healthy controls^113,114^. An increase of genus *Ruminococcus* was also shown upon treating antidepressants or probiotics on the stress-induced mice^115^, and the negative association with the amyotrophic lateral sclerosis patients was reported by another gut microbiome study^116^. Contrastingly, another report found that *Ruminococcus flavienciens* was able to diminish the effects of antidepressants on the depressive-like behavior in mice^117^. These inconsistent results could be attributed by the differential influences of *Ruminococcus* species, thus the species level investigation is suggested to more clearly understand the association.

In this study, *R. albus* was increased 2.93-fold in mice supplemented with EG007 and had a significantly positive correlation with the spatial learning and memory (r = 0.63), and it was previously demonstrated that intestinal *R. albus* had neuroprotective effects by reducing oxidative stress and increasing BDNF level in brain^118^. Also, the results are paralleled by the finding that *R. albus* was not found in the stools of children with ASD, while a significant abundance of *R. albus* was found in control children^110^, suggesting its positive role. Any findings regarding *R. bicirculans* (increased 2.99-fold, r = 0.75) and *R. champanellensis* (increased 2.90-fold, r = 0.75) have not previously been reported so far. In addition to exerting various health benefits in the colon and the peripheral tissues, SCFAs play a pivotal role in the MGB crosstalk through crossing the blood–brain–barrier (BBB) and influencing the enteric nervous system activity^119^. *Ruminococcus* genus has been identified as one of the bacterial communities responsible for intestinal production of SCFAs^109^. SCFAs mainly consist of butyrate, propionate, and acetate^120^, and it has been suggested that each of them exerted a different function on the nervous system physiology. While butyrate is known as involved in histone deacetylase inhibition, anti-depressant effects, and regulating BBB and gut permeability^109,121^, propionate was reported to cause developmental delay or seizures^122^ and alter dopamine, serotonin, and glutamate systems in a manner similar to that observed in ASD^123,124^. Previous studies have also found that a subset of ASD patients has high levels of propionate-producing species^114,125,126^. Acetate showed an anti-neuroinflammatory effect against AD via upregulating G-protein-coupled receptor 41 and suppressing ERK/JNK/NF-kB signaling pathways^127^, and the acetate level was significantly downregulated in the AD model^128^. Thus, it is speculated that the different fermentation products of *Ruminococcus* species potentially resulted in the different associational pattern as *R. albus* and *R. bicirculans* are known to produce acetate and formate from carbohydrates^118,129^, whereas other species such as *Ruminococcus obeum* and *Ruminococcus bromii* were reported to associate with propionate production^130,131^. Future biochemical studies are required to provide further insights into the underlying molecular mechanisms through which these species contribute to the brain function, and also cognition-associated microbiota affected by EG007 supplementation presented in this study will serve as preliminary data for future associative studies.

## Conclusion

In summary, our study observed that 8-weeks consumption of EG007 markedly promoted short-term learning and memory skills in healthy young male mice. By constructing complete genome sequence of EG007, we identified genes encoding various metabolites that might have contributed to the enhancements in gut microbiota and brain function, including bacteriocin-coding genes and the genes encoding glutamate decarboxylase (*gadB*) and glutamate/GABA antiporter (*gadC*), which are necessary for GABA production. Further, as a result of gut microbiota community analysis utilizing 16S–23S sequencing, we gained insights into EG007-mediated compositional changes at the species level. Key signature alterations were observed including down-regulation of inflammation or infection-related species and enrichment of species, previously reported to have an association with the CNS functions such as *P. faecium* and *Ruminococcus* species. Moreover, correlation analysis highlighted that eight significantly modulated microbiota upon EG007 supplementation were also closely related to the cognition traits, which have not been reported so far. Collectively, the results above suggested that *S. thermophilus* EG007 was an important exogenous factor that can improve cognitive functions.

Although we obtained meaningful results, there are still several limitations that needed to be mentioned. For behavior tests, the forced alternation test could be performed before the spontaneous alternation test. Although there were two weeks of gap between the two tests, measuring the natural curiosity against the novel arm would be more accurate if the mice did not have any prior information about the space. Also, this study does not provide direct evidence of memory improvement linking the MGB axis. Therefore, further biochemical studies identifying gut-brain related biomarkers are needed to elucidate the mechanisms of action underlying the cognition-enhancing effects of EG007 or the microbiota modulated by EG007.

Despite these limitations, this is the first study examining the effects of a single *S. thermophilus* strain supplementation on brain function and also one of few studies focused on the effects in young healthy subjects, rather than populations with neurological disorders or age-related cognitive declines. Besides, in the present study, nanopore sequencing was employed in the genomics and metagenomics analysis to investigate the probiotics and psychobiotics properties. This application of long-read sequencing technology had a critical role particularly in providing more accurate and higher resolution of taxonomic inferences of healthy gut microbiota which have more complex characteristics, thereby revealing more detailed *S. thermophilus*-derived changes and microbiota-cognition relationships at the species level. The genomic features and gut microbial modulations revealed in this study will serve as preliminary data for future associative studies and human clinical trials and will provide a potential of *S. thermophilus* in assisting cognitive function enhancements by possible MGB axis modulating capability, which can be applied for the more general population of healthy and young people.

## Methods

### Animals and housing

All animal experiments were performed following NIH Guide for the Care and Use of Laboratory Animals approved by the Institutional Animal Care and Use Committee of Seoul National University (approval number: SNU-190607-4-3). The study is reported in accordance with ARRIVE guidelines. Male SPF C57BL/C mice (n = 36, 4 weeks old on arrival) were purchased from the Youngbio (Seongnam, Republic of Korea). Mice were housed in groups of four littermates per cage (33 cm × 15 cm × 13 cm, L × H × W) in the animal facility at the Seoul National University and allowed to acclimatize for one week prior to dietary administration. Cages of mice were randomly divided into three groups (ST, LP, and Control, *n* = 11–12/group). All mice were provided with standard chow diet in pelleted form, and the groups were provided with sterilized water (Control group) or sterilized water supplemented with 1.08 × 10^10^ CFU of *S. thermophilus* EG007 (ST group) or 3.12 × 10^10^ CFU of *L. plantarum* A003F7 (LP group) ad libitum throughout the whole study. They were maintained in a ventilated room under standard controlled laboratory conditions: a 12-h light/dark cycle (lights on at 7:00 am), a temperature of 22 ± 2 °C with 50 ± 5% humidity. Cages were changed once a week by the same experimenter.

### Study design

The graphical experiment design is presented in Fig. 1. After a week of acclimatization to the animal facility, 5-weeks-old mice of experimental groups (ST; n = 12 and LP; n = 11) were provided EG007 or A003F7, respectively, in drinking water daily ad libitum for 8 weeks, and control group (control; n = 12) were given normal drinking water ad libitum throughout the study. All mice were weighed once weekly and water consumption was monitored every day. From week 5 (9 weeks of age) onward, all mice were subjected to the same series of behavior tests to assess their cognitive performance, the Y-maze test (spontaneous alternation and forced alternation) for short-term spatial memory, the novel object recognition test for short-term nonspatial memory, and the passive avoidance test for long-term associative memory. Tests were ordered from the least to the most stressful task, including resting days between tests, to avoid carryover effects. All behavior tests were performed between 1 and 6 pm in the active phase of the animal under dim red light to reduce anxiety in the animals. On the day of testing, mice were transferred in their original cages to the behavior testing room 2 h before the test to minimize stress. Animals were tested one at a time in a counterbalanced fashion regarding testing order and under the same conditions, and all apparatus used in the testing were thoroughly cleaned with 70% EtOH between animals or sessions to remove odors. Test sessions were recorded using camera mounted facing straight down, and two independent experimenters blinded to conditions manually scored behaviors of the videos. All mice were sacrificed 5 days following the last test (week 9).

### Probiotic administration

Both strains (EG007 and A003F7) were isolated from fermented dairy products in the present laboratory. In this study, to avoid stress in the animals caused by daily use of oral gavage, mice were administered probiotics, dissolved in drinking water. The water containing EG007 or A003F7 was freshly prepared just before administration from bacteria grown overnight and changed every morning on a daily basis throughout the study (8 weeks). For the control group, sterile water was provided and changed every morning as well. Each probiotic strain was cultured aerobically in de Man, Rogosa, Sharpe (MRS) media at 37 °C for 9 h each, harvested by centrifugation at 4000 rpm for 10 min, and washed twice with sterile saline. The collected cells were resuspended in the same volume of sterile water at an average concentration of 1.45 × 10^9^ CFU/ml (EG007) and 3.73 × 10^9^ CFU/ml (A003F7). Water intakes were recorded every day throughout the experiment. The viability of probiotics and the final dose in the drinking water have been confirmed by plating resuspended water on the MRS plates every day. The final concentration ingested by each mouse was 1.08 × 10^10^ CFU (EG007) and 3.12 × 10^10^ CFU (A003F7) per day based on plating results (Supplementary Fig. 1).

### Y-maze test

Two versions of Y-maze tests were performed: Spontaneous Alternation test and Forced Alternation test. The Y-maze apparatus (Jeung Do Bio & Plant CO., LTD, Seoul, Korea) was consisted of three Y-shaped white plastic arms symmetrically disposed at 120° angles from each other (40 cm long, 5 cm wide, and 10 cm high) and used in both tests. To differentiate three arms, ground in the testing room around each arm was marked, and they were kept in place throughout the testing period. Y-maze test is generally regarded as a measure to evaluate short-term spatial working memory in mice^132,133^ based on their innate tendency to explore novel space, and it reflects the functions of prefrontal and hippocampal systems^134^. For Spontaneous Alternation, mice were randomly placed at the end of one of the arms to avoid placement bias, facing the wall, and allowed to freely explore the three arms for 6 min. The sequence of arm entries and the total number of arm entries were recorded to measure the alternation score. A spontaneous alternation is defined as entering a different arm in three consecutive entries (e.g., ABC, CBA, or BCA but not BAB), and the alternation score was calculated as follows: $\text{Alternation score }\left(\text{\%}\right)= \frac{(number \,of \,alternations)}{(total \,number\, of\, arm\, entries-2)} \times 100$. The total number of arm entries was also used as a locomotor activity indicator.

Forced Alternation test consisted of the training phase, intertrial interval, and testing phase^135^. During the training phase, access to one of the arms (novel arm) was blocked by a white barrier, and mice were placed in the start arm and allowed to explore the two open arms of the maze (start and familiar arm) for 5 min. After 1 h of an intertrial interval, the testing phase was performed with the barrier removed this time. The mice were again placed in the Y-maze in the same start arm, and all three arms (start, familiar, and novel arms) are opened to explore for 5 min. During the testing phase, the time taken for mice to enter the novel arm (latency), the percentage of time spent in the novel arm, and the percentage of entries into the novel arm were calculated to determine the tendency to explore the novel arm. For both of the tests, arm entries were counted when all four paws entered into the 1/3 point of the arm, and if a mouse climbed the maze wall, it was immediately returned to the abandoned spot.

### Novel object recognition test

Novel object recognition (NOR) test was performed to evaluate short-term non-spatial memory of mice, exploiting their innate tendency to explore a novel object^136^. It reflects the dorsal hippocampal function which is associated to the ability to recognize a familiar object^137^. This test consisted of three phases: training phase, intertrial interval, and testing phase. Prior to the test, several objects were tested to avoid object bias, and objects with equal preference were selected for the NOR test. The objects used were of different shape and color but of equivalent texture and volume (size 2–3 × 4–5 cm): two star-shaped purple plastic toys and two triangle-shaped green plastic toys. The object recognition testing arena (Jeung Do Bio & Plant CO., LTD, Seoul, Korea) was made of a white plastic open field (40 cm × 40 cm × 40 cm, L × W × H). During the training phase, two identical objects were placed in opposite quadrants of the testing arena (e.g., one in the NW corner and one in the SE corner), and mice were placed facing the wall in the corner, equidistant from both objects and allowed 10 min for free exploration. After 1 h of an intertrial interval, in the testing phase, mice were positioned again in the same location of the arena, facing the wall, in which one of the two identical objects was substituted with a novel object. For each mouse, objects were placed on the same diagonal location as used in the training phase, and mice were given 5 min to freely explore the objects. The diagonal location and the position of the novel object (left or right) was randomly assigned and counterbalanced between each mouse and conditional group. Preference for a novel over a familiar object was determined by measuring the amount of time explored the novel object relative to the familiar one, and the results were expressed as a discrimination ratio. The discrimination ratio was calculated as the percentage of novel object recognition time as follows: $\text{Discrimination ratio }= \frac{{Time}_{novel}}{{Time}_{novel }+ {Time}_{familiar} } \times 100$. Exploratory behavior was counted when a mouse approached an object with its nose pointed towards the object, with active sniffing or vibrissae sweeping within 2.5 cm of the object. A mouse climbing on top of the object or approaches without paying attention was not considered.

### Passive avoidance test

The passive avoidance (PA) test was performed to evaluate long-term associative learning and memory^138^ of mice through Pavlovian fear conditioning, based on the innate aversion to the brightly illuminated areas. We used a step-through shuttle-box (Jeung Do Bio & Plant CO., LTD, Seoul, Korea) consisted of two chambers (white and black chambers) separated by a sliding door, and the floor of the chambers consisted of stainless-steel rods (2 mm diameter) spaced 1-cm apart. The white chamber (21 × 21 × 30 cm) was illuminated, while the black (21 × 21 × 30 cm) remained dark. During the training phase, mice were placed in the illuminated chamber with the door closed, and the door was raised noiselessly when the mice were facing away from it. When the mice entered the dark chamber, the door was closed and a mild foot shock of 0.25 mA was delivered for 3 s through the stainless-steel rods using a shock generator. Mice were remained in the dark chamber for 30 s after shock, and then returned to the home cage. During the process, mice learned the associative rule that a black chamber is equal to shock^139^. 24 h post training, the testing phase commenced. Trained mice were again placed in the illuminated chamber, and the same procedure was conducted except for the foot shock. In both phases, the latency time to entering the dark chamber with all four paws was measured with a cut-off duration of 300 s. The counting started immediately as the door fully raised. If a mouse did not enter the dark chamber within 300 s, 300 s latency was assigned and the mouse was returned to its home cage.

### Statistical analysis for mice experiments

All data from the behavior experiments were expressed as mean ± standard error of the mean (SEM). After assessing the normality and homoscedasticity according to Shapiro–Wilk test and F-test, differences between the mean values were assessed using one-way analysis of variance (ANOVA) or Kruskall–Wallis test, where appropriate, followed by the Tukey’s *post-hoc* test. Correlation was assessed by Pearson’s statistics. The differences were considered statistically significant when Benjamini–Hochberg False Discovery Rate (FDR) adjusted *P*-value < 0.05.

### Fecal DNA extraction and metagenomic sequencing of 16S–23S rRNA

At the end of the animal study, stool samples were collected from individual mice in a sterile tube and immediately frozen and stored at − 80 °C. Eight samples (four randomly chosen from each group) were subjected to lysis and cell disruption by Zirconia/Silica Beads and proteinase K (Biospec Products, Seoul, Korea), and genomic DNA was extracted using AccuPrep® Stool DNA Extraction Kit (Bioneer, Daejeon, Korea). Isolated gDNA was quantified and qualified by gel electrophoresis, 260/280 nm absorbance ratio and Quant-iT™ PicoGreen™ dsDNA Assay Kit (Invitrogen, Carlsbad, CA, USA). The sequencing library was constructed by target-specific PCR amplification of the 16S–23S region of the ribosomal operon (~ 4200 bp), followed by clean-up with Agencourt AMPure XP beads (Beckman Coulter, Brea, CA, USA). The resulting libraries were prepared using the ONT 1D Ligation Sequencing Kit (SQK-LSK109; Oxford Nanopore Technologies) and loaded onto the Flongle Flow Cell (FLO-FLG001; Oxford Nanopore Technologies) according to the manufacturer’s protocol. Instead of multiplexing, each sample library was sequenced on a single Flongle flow cell in order to avoid read loss from demultiplexing step (1 fecal sample library per 1 Flongle flow cell). Once the libraries were loaded, they were sequenced on the MinION MIN-101B, and MinKNOW software 19.12.5 (Oxford Nanopore Technologies) was used for data acquisition.

### Microbial community analysis and statistical analysis

After the eight sequencing runs were completed, fast5 files were base-called using Guppy GPU basecaller (v4.2.2), and Porechop (v0.2.4, https://github.com/w1bw/Porechop) was used to remove the sequencing artifacts and chimeric reads. After the trimming step, reads were filtered by size to retain sequences with 3500 to 5000 bp length. To assign taxonomy, the processed reads from each sample were aligned against MIrROR database (mirror.snu.ac.kr; https://github.com/seoldh/MIrROR) which was constructed in our accompanying study using Minimap2 aligner (v2.17)^140^ with ‘–secondary = no’ option. The MIrROR database contains sequences of 97,781 full 16S–23S rRNA operon sequences of the 43,653 bacterial strains representing 9485 species, annotated according to GTDB taxonomy. We kept only those reads that aligned to the reference with a block ≥ 2500 bp and the number of matching bases ≥ 1500 bp. For reads that hit more than one reference sequence, only the alignments with the highest alignment score (AS score; Smith–Waterman alignment score) were retained. Bacterial diversity was calculated by alpha-diversity (Shannon’s index, Simpson index, Observed OTUs, ACE) and beta-diversity (Weighted and Unweighted Unifrac and Bray–Curtis dissimilarity). For detection of differentially abundant microbiota, a negative binomial based generalized linear model (GLM) was employed using EdgeR R package^141^ with trimmed mean of M-value (TMM) normalization method for consideration of different read production.

### DNA extraction and whole genome sequencing of *S. thermophilus*

EG007 was grown aerobically in MRS broth for 9 h at 37 °C. Genomic DNA was extracted using PureHelix™ Genomic DNA Prep Kit (Solution Type)-Bacteria (NanoHelix, Daejeon, Korea), and isolated gDNA was quantified and qualified using gel electrophoresis, 260/280 nm absorbance ratio and Quant-iT™ PicoGreen™ dsDNA Assay Kit (Invitrogen, Carlsbad, CA, USA). The library was prepared using the ONT 1D ligation Sequencing kit (SQK-LSK109). The resulting library was loaded onto the Flongle Flow Cell (FLO-FLG001, R9.4.1) and sequenced on the MinION MIN-101B and MinKNOW (19.06.8).

### Genome assembly, annotation, and analysis

Base-calling was carried out using Guppy GPU basecaller (v4.2.2). Sequencing artifacts and chimeric reads were removed using Porechop (v0.2.4, https://github.com/w1bw/Porechop), and reads with a quality score below 10 were filtered out using Nanofilt (v2.7.1)^142^. Genome assembly was conducted using CANU (v2.1.1)^143^ with genomeSize = 1.9 Mb parameter. In order to increase consensus accuracy, multiple rounds of polishing with racon (v1.4.17)^144^ and medaka (v1.0.3) (https://github.com/nanoporetech/medaka) were conducted until no additional correction could be made. Finally, the assembled reads were circularized using Circlator^145^. Genome annotation was performed using the Prokka (v1.14.6) with-rfam option to enable a search for ncRNA, and automatic annotation results were also collected from Rapid Annotation using Subsystem Technology (RAST). The predicted CDSs were then assigned in different clusters of orthologous group (COG) functional categories using eggnog-mapper tool (v2.0.1b)^146^, and COGs were retrieved using a bash script—eggnog-mapper_COGextraction (https://github.com/raymondkiu/eggnog-mapper_COGextraction). Genomic Islands (GIs) were detected through the IslandViewer4 web-based resource^147^, and the CRISPRs were identified with CRISPRCasFinder web tool^148^. For putative prophage detection, PHASTER was utilized^147^. Antibiotic resistance genes and virulence factor-related genes were predicted using CARD^149^, ResFinder^150^, and VFDB^151^. Lastly, ISFinder^152^ was used to identify insertion sequences, and BAGEL4^153^ was utilized to identify potentiality to produce bacteriocins. For identification of stress tolerance related genes, sequence information for different probiotic genes^154^ were obtained from the NCBI database and used for BLAST-based sequence similarity search.

### Phylogenetic analysis

The phylogenetic tree was constructed with 849 1-to-1 orthologous genes in 65 publicly available complete genomes of *S. thermophilus* and EG007 identified by PorthoMCL^155^. The genes were individually aligned using MUSCLE^156^ and refined using Gblock^157^. Concatenated sequences were subjected to construct a neighbor-joining tree with 1000 bootstrap replicates using MEGA-X^158^. *Streptococcus salivarius* L25 genome was used as an outgroup.

## Supplementary Information

Supplementary Figures.

Supplementary Table 1.

Supplementary Table 2.

## Data Availability

The datasets generated during the current study are available in the NCBI BioProject accession no. PRJNA698776 and PRJNA834938.
