# Supplementary material for: Investigation of memory-enhancing effects of Streptococcus thermophilus EG007 in mice and elucidating molecular and metagenomic characteristics using nanopore sequencing
Source: Sci Rep. 2022 Aug 2;12:13274. doi: 10.1038/s41598-022-14837-z (PMC9346115; doi:10.1038/s41598-022-14837-z)
Supplement: Supplementary file 1 — Supplementary Figures. [file 41598_2022_14837_MOESM1_ESM.pdf]

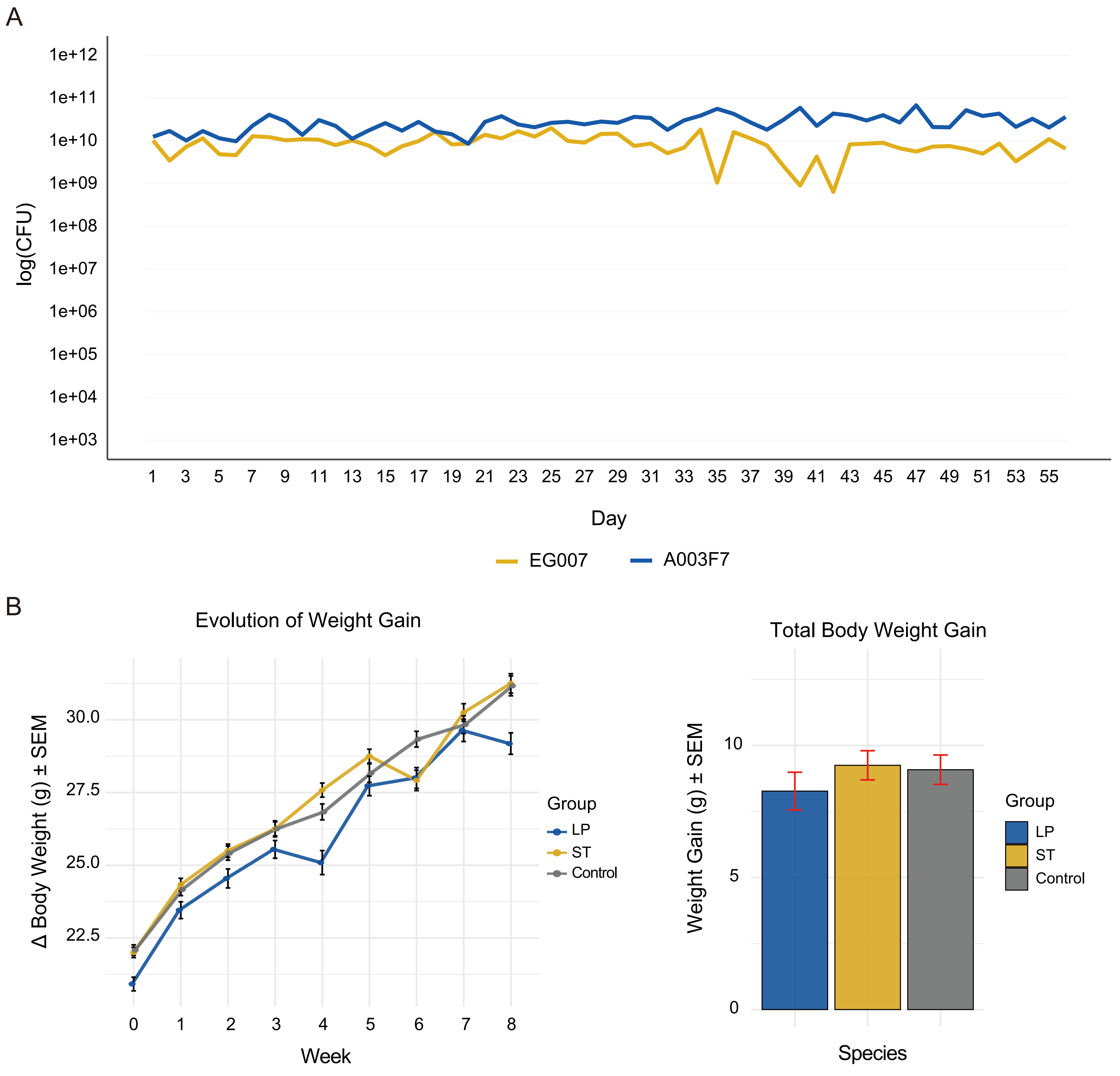

Supplementary Figure 1. Average daily dose of probiotics and effects of probiotics supplementation on the weight. (A) Average daily dose of probiotics. (B) Evolution of weight gain and total body weight gain for eight weeks.

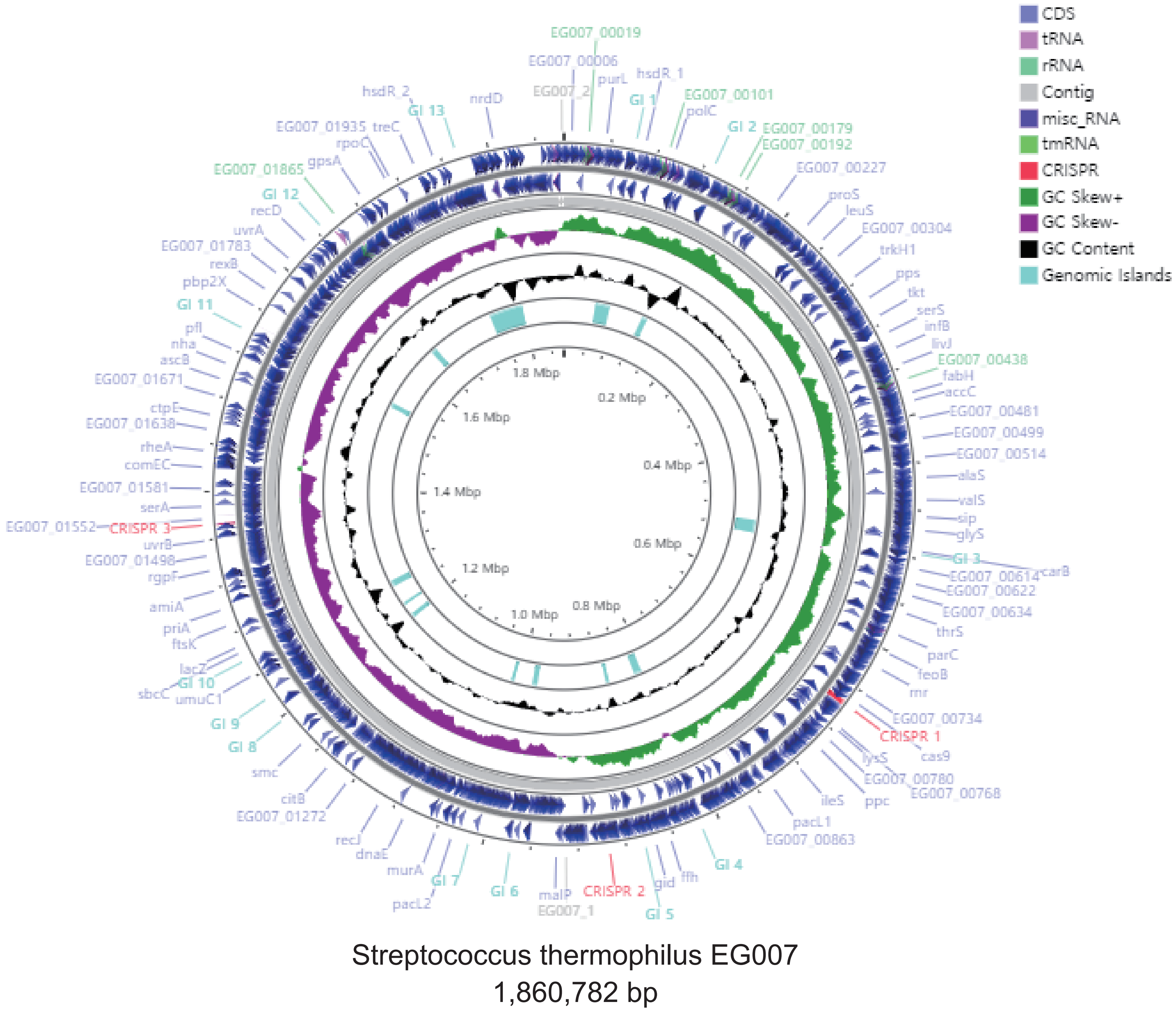

Supplementary Figure 2. Circular genomic structure of S. thermophilus EG007.

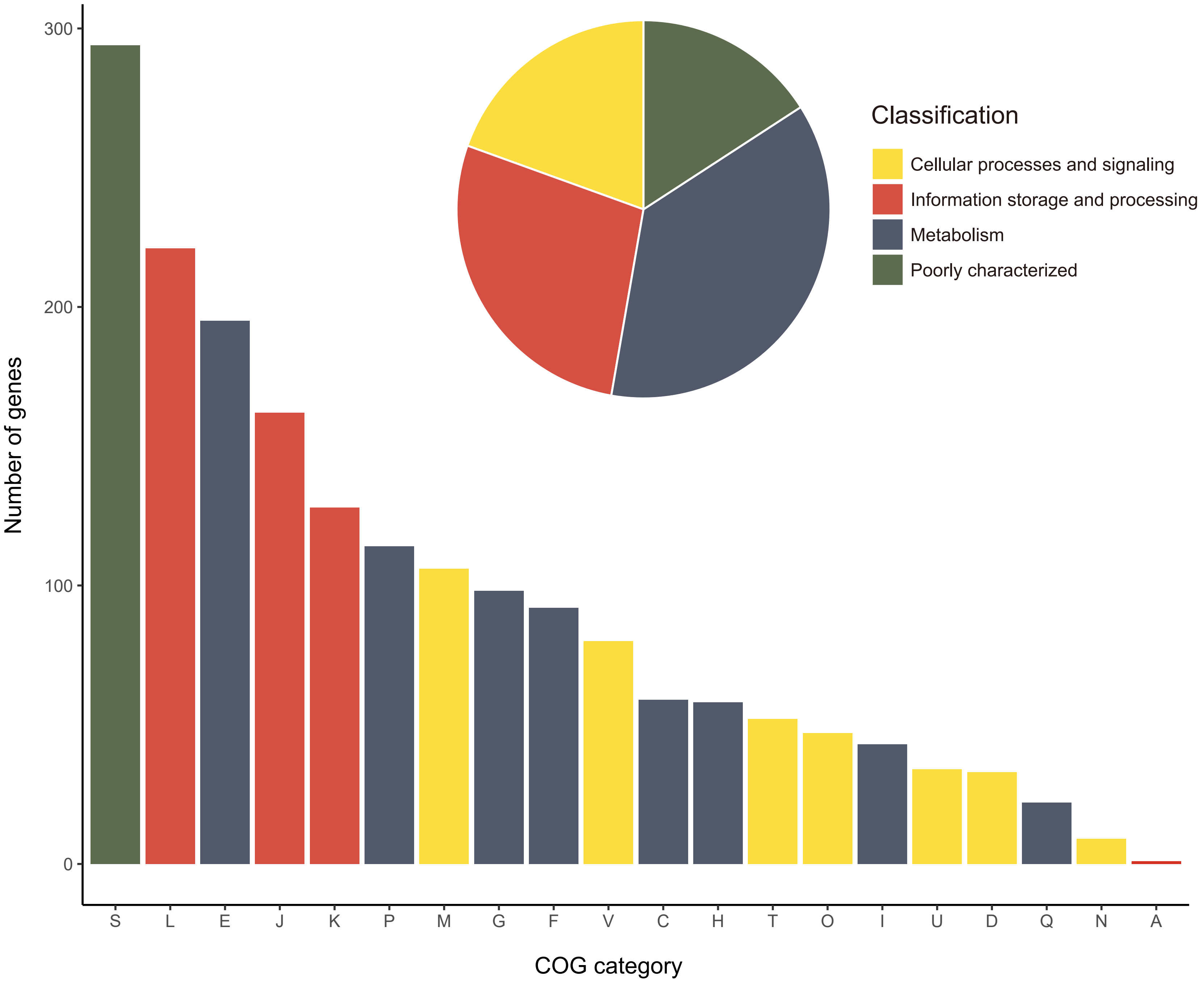

Supplementary Figure 3. Distribution of clusters of orthologous group (COG) functional categories.

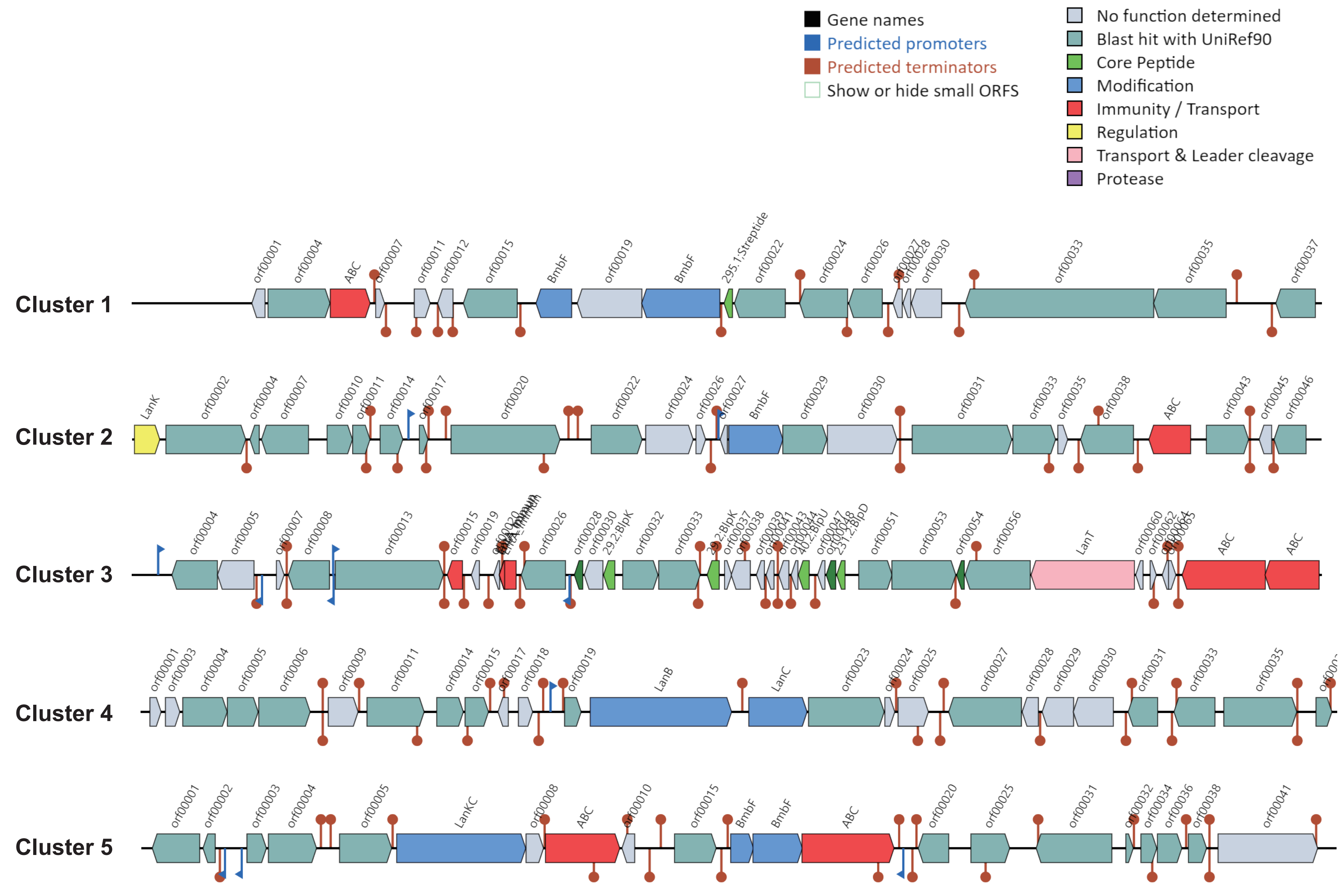

Supplementary Figure 4. Genetic organization of bacteriocin clusters detected in S. thermophilus EG007 genome.

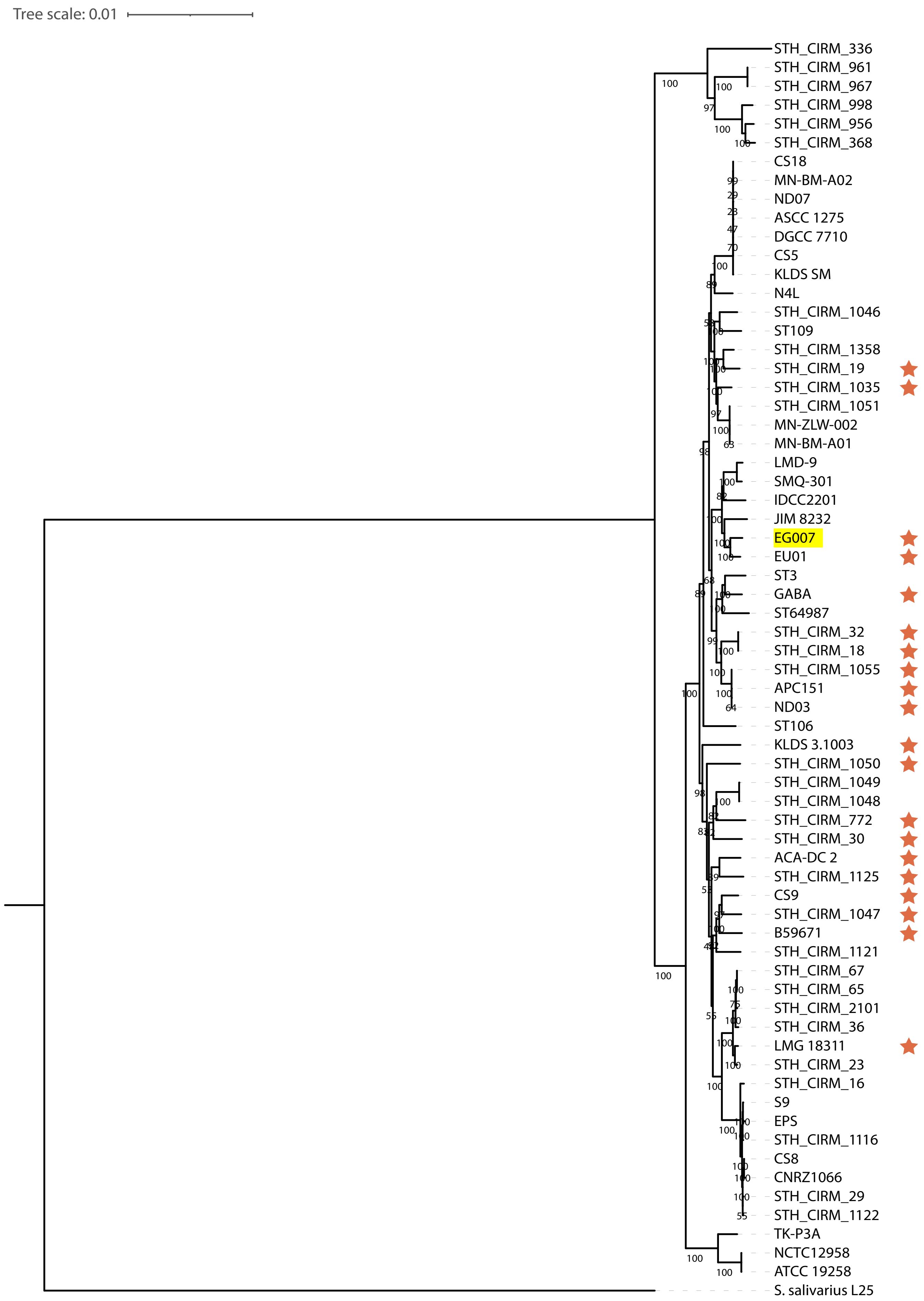

Supplementary Figure 5. Phylogenetic tree of publicly available S. thermophilus complete genomes based on 849 orthologous genes and distribution of GABA operon.

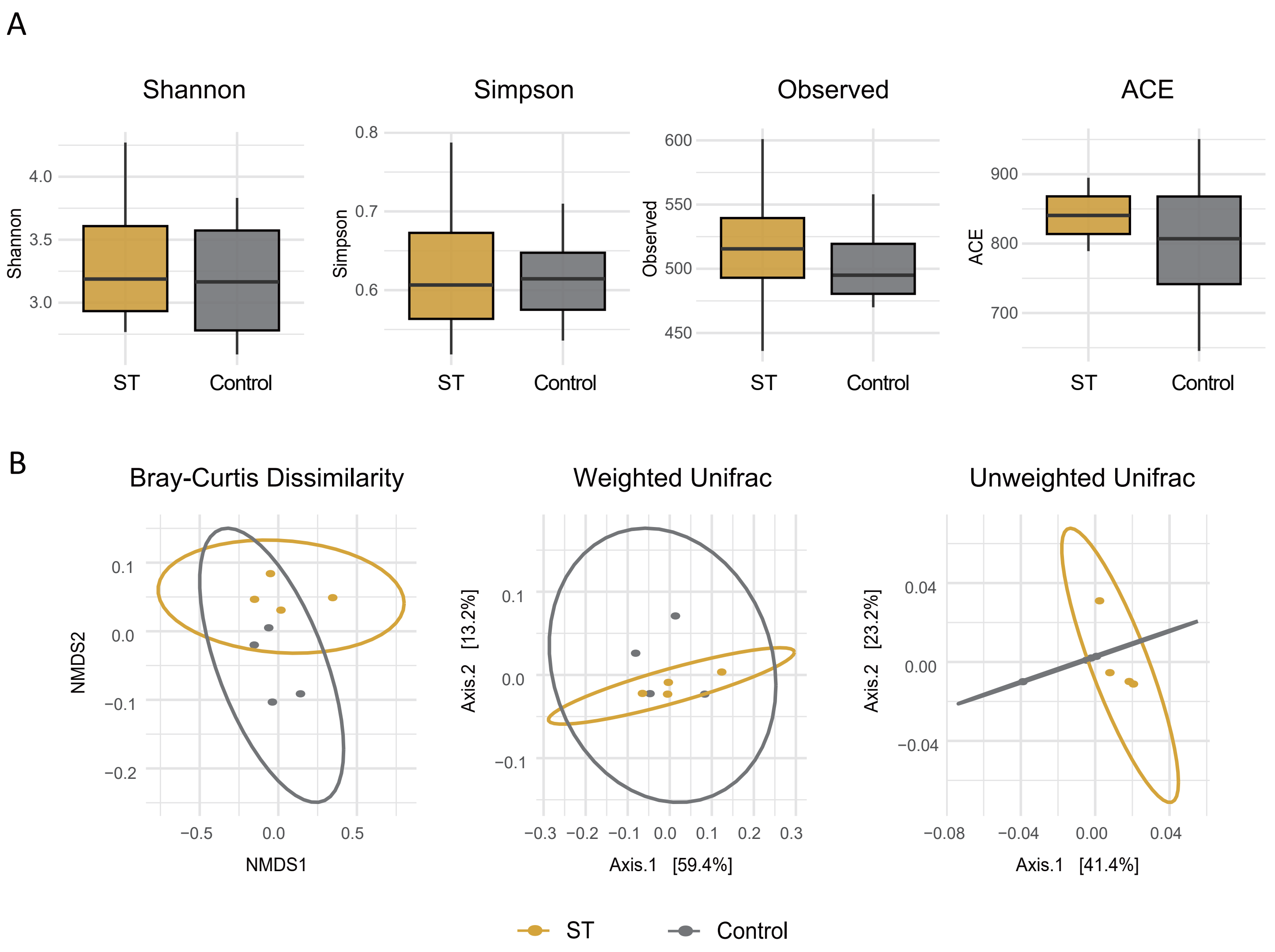

Supplementary Figure 6. Diversity of fecal sample microbiota. (A) alpha diversity. (B) beta diversity.

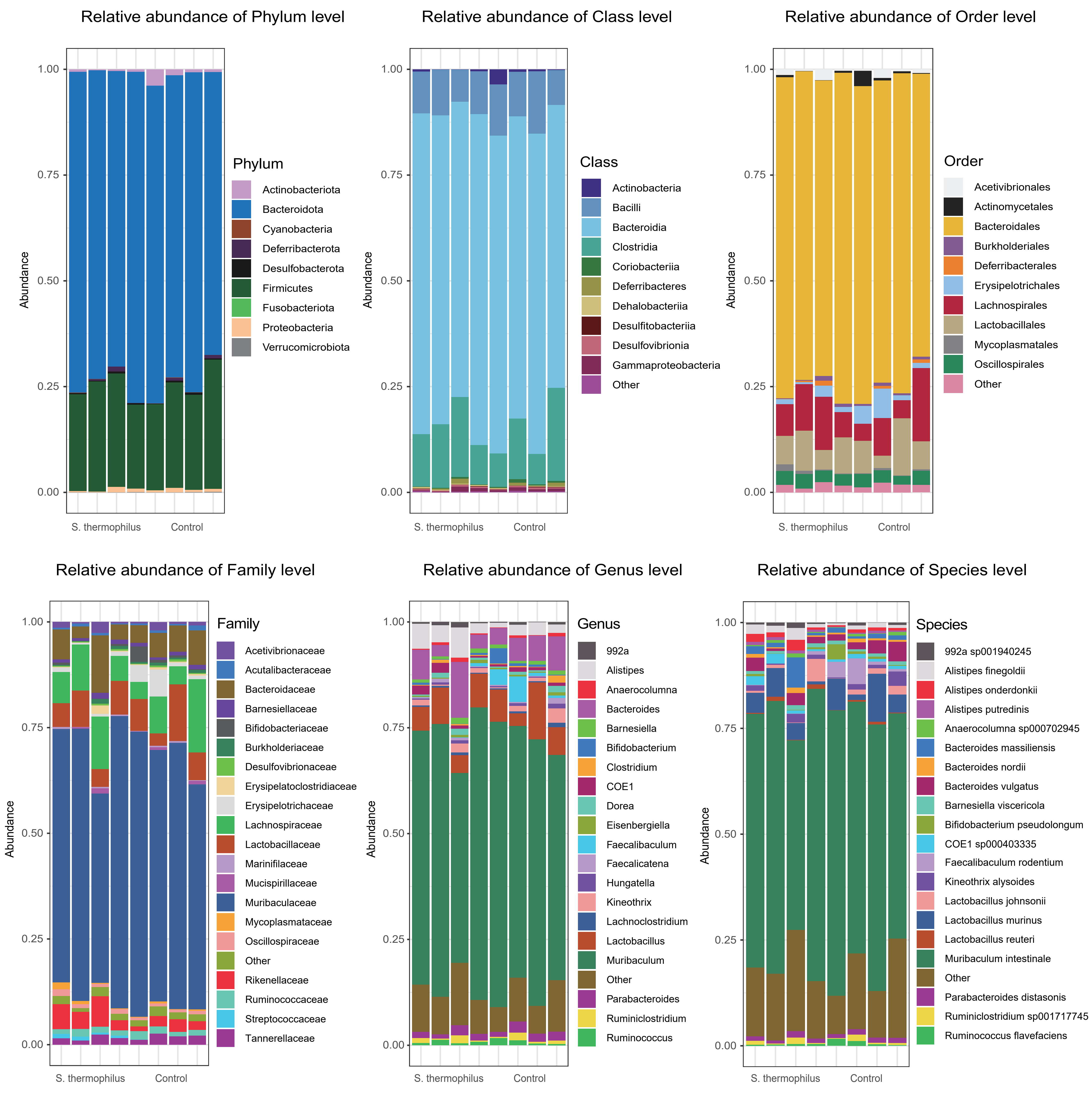

Supplementary Figure 7. Composition of gut microbiota at hierarchical taxonomy levels.
